# STaRRRT: a table of short tandem repeats in regulatory regions of the human genome

**DOI:** 10.1186/1471-2164-14-795

**Published:** 2013-11-15

**Authors:** Katherine A Bolton, Jason P Ross, Desma M Grice, Nikola A Bowden, Elizabeth G Holliday, Kelly A Avery-Kiejda, Rodney J Scott

**Affiliations:** 1 Centre for Information-Based Medicine, Hunter Medical Research Institute, Newcastle, NSW, Australia; 2 Priority Research Centre for Cancer, School of Biomedical Sciences and Pharmacy, University of Newcastle, Newcastle, NSW, Australia; 3 Preventative Health National Research Flagship, CSIRO, North Ryde, NSW, Australia; 4 Animal Food and Health Sciences, CSIRO, North Ryde, NSW, Australia; 5 School of Medicine and Public Health, University of Newcastle, Newcastle, NSW, Australia; 6 Hunter Area Pathology Service, Hunter New England Health, Newcastle, NSW, Australia; 7 Head of the Discipline of Medical Genetics, School of Biomedical Sciences and Pharmacy, Faculty of Health, University of Newcastle, University Drive, Callaghan, NSW 2305, Australia

**Keywords:** Short tandem repeats, STR, Microsatellites, Simple sequence repeats, SSR, Promoter, Regulatory region, Neurological disease, Neural genes, Evolution

## Abstract

**Background:**

Tandem repeats (TRs) are unstable regions commonly found within genomes that have consequences for evolution and disease. In humans, polymorphic TRs are known to cause neurodegenerative and neuromuscular disorders as well as being associated with complex diseases such as diabetes and cancer. If present in upstream regulatory regions, TRs can modify chromatin structure and affect transcription; resulting in altered gene expression and protein abundance. The most common TRs are short tandem repeats (STRs), or microsatellites. Promoter located STRs are considerably more polymorphic than coding region STRs. As such, they may be a common driver of phenotypic variation. To study STRs located in regulatory regions, we have performed genome-wide analysis to identify all STRs present in a region that is 2 kilobases upstream and 1 kilobase downstream of the transcription start sites of genes.

**Results:**

The Short Tandem Repeats in Regulatory Regions Table, STaRRRT, contains the results of the genome-wide analysis, outlining the characteristics of 5,264 STRs present in the upstream regulatory region of 4,441 human genes. Gene set enrichment analysis has revealed significant enrichment for STRs in cellular, transcriptional and neurological system gene promoters and genes important in ion and calcium homeostasis. The set of enriched terms has broad similarity to that seen in coding regions, suggesting that regulatory region STRs are subject to similar evolutionary pressures as STRs in coding regions and may, like coding region STRs, have an important role in controlling gene expression.

**Conclusions:**

STaRRRT is a readily-searchable resource for investigating potentially polymorphic STRs that could influence the expression of any gene of interest. The processes and genes enriched for regulatory region STRs provide potential novel targets for diagnosing and treating disease, and support a role for these STRs in the evolution of the human genome.

## Background

Tandem repeats (TRs) are stretches of DNA that contain nucleotide patterns repeated adjacent to one another and are common throughout the human genome [1]. TRs are classified by repeat unit length into further categories including microsatellites, or short tandem repeats (STRs), which are repeats with a unit length of less than 10 nucleotides or base pairs (bp). TRs display a non-random distribution and a particular bias in location to genic and regulatory regions [2,3]. In humans, approximately 17% of genes contain TRs within their coding regions [4]. In yeast (*Saccharomyces cerevisiae*), approximately 25% of all gene promoters contain at least one tandem repeat (TR), many of these TRs consisting of short, AT-rich sequences and the distribution of TRs in human gene promoters is similar [5,6].

TRs have a propensity to mutate and become polymorphic by expansion or contraction in the number of repeat units. This may be due to slippage during DNA replication, through unequal crossing-over during recombination, or by imprecise repair of double-strand DNA breaks [7-9]. TRs exhibit mutation rates around 10 to 10^5^-fold higher than average rates for non-repeated DNA in other parts of the genome [7,10-12]. Such polymorphic TRs are often described as variable number of tandem repeats (VNTR). The frequency of TR mutations is dependent upon the length of the repeat unit (known as the “period”), the number of repeat units, and the percentage match to the consensus sequence or “purity” of the repeat tract [4,13]. The number of repeat units and purity of the repeat tract are the most important predictors for repeat variability, with an increase in the number of repeats and/or purity resulting in a higher propensity to be polymorphic [13,14]. Naslund *et al.* (2005) found that doubling the repeat unit number corresponded to a 15-fold increase in the likelihood of the repeat being polymorphic and for each 10% increase in repeat purity, an 18-fold increase in likelihood of polymorphism resulted.

STRs are a common source of genetic variation in promoter regions and alleles can be highly variable in length. In humans, the rate of STR length polymorphism within 1 kb upstream of the transcription start site (TSS) is over 12-fold higher than in exonic regions, 1.5-fold higher than in untranslated regions (UTRs) and almost comparable to the rate in intragenic and intronic regions [15]. Despite this hyper-variability, there is also evidence for promoter localised STRs being evolutionarily conserved [6]. The conservation rate of STRs is dependent upon the proximity to the TSS, with closer STRs more likely to be conserved [16].

Polymorphic TRs can affect transcription by a number of means. Length polymorphism has consequences for transcription, with TR-containing promoters showing significantly higher rates of transcriptional expression divergence [5]. In yeast, it is known that nucleosome position is inversely correlated with tandem repeat positions with nucleosome depletion being especially pronounced around AT-rich repeats [5]. In addition, altering the length of TRs in promoter regions directly affects the local chromatin structure resulting in altered transcriptional activity and gene expression [5,17]. Further, potential sites of Z-DNA are enriched at the promoter and 5’-end of human genes [18] and Z-DNA, which expels bound nucleosomes, is more likely to form where the AC/GT dinucleotide repeat is present [19]. Combined, the exceptionally high polymorphism rate, evolutionary conservation around the TSS and evidence for transcriptional regulation suggests that promoter STRs are functional and may be an important source of rapid evolutionary change. If so, STRs should also be associated with disease.

Polymorphic TRs are implicated in more than 40 neuromuscular and neurodegenerative diseases, such as spinobulbar muscular atrophy [20] and Huntington’s disease [21]; as well as other complex disorders such as anxiety [22], mental retardation [23] and diabetes [24,25]; and several cancers, such as colorectal [26,27] and prostate cancer [28-30]. In the regulatory region, polymorphic STRs in the *FLI1, ECE-1c* and *CD30* gene promoters have been associated with lupus [31], Alzheimer’s disease [32] and primary cutaneous lymphoproliferative disorders [33], respectively.

While there is mounting evidence that STRs are an important class of genetic variation with links to disease phenotypes and evolution of the human genome, their use in genetic studies has reduced with the advent of massively parallel single nucleotide polymorphism (SNP) analysis and genome-wide association studies (GWAS) [34,35]. Compared with SNPs, STRs show extremely rapid evolution, indicative of increased variability between individual sub-populations. The observed enrichment of STRs in genic and regulatory regions [4] also suggests potentially larger phenotype effects than many common SNPs. Hypervariable STRs in regulatory regions may explain some of the missing heritability unaccounted for by GWAS of complex disease [13,36,37]. From a human genetics perspective, this untapped source of regulatory STR variation could be important and also complementary to GWAS studies. Increasing interest over the past decade in the noncoding regions of the human genome, which has been described as “the control architecture of the system” [38], further highlights the important role that variation in these regions plays. Considering the influential role of STRs in regulating gene expression, the importance of this source of genetic variation has been over-looked.

There is currently no catalogue or easy to use resource available for studying STRs in the regulatory regions of human genes. This study aimed to identify, characterise and compare STRs in the upstream regulatory region of human genes on a genome-wide scale and establish a resource to allow the interrogation of STRs in this region. By screening the entire human genome, using Tandem Repeat Finder [39], SQL code and the UCSC Genome Browser [40], for STRs present in a 3 kilobase region at the 5’-end of all human genes, we have identified 5,264 STRs across 4,441 genes. The information describing the location and characteristics of these STRs is presented in the Short Tandem Repeats in Regulatory Regions Table, or STaRRRT (available at http://www.newcastleinnovationhealth.com.au/STaRRRT). This resource is suitable for researchers with limited bioinformatics experience who are interested in specific STRs, genes or phenotypes. We have identified a unique signature of STR enrichment in the regulatory regions of human genes which is most pronounced within neural genes, and calcium signaling and neurological pathways. This paper presents the findings from investigations of the distribution and abundance of STRs in the 5’ regulatory region of human genes, highlighting the importance of STRs in neurological pathways and in recent evolution of the human genome.

## Results

### STaRRRT is a comprehensive, user-friendly resource with wide application

**Figure 1:**
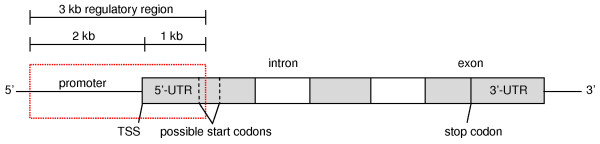
**Location of the regulatory region analysed in a representative human gene.** The location of the 3 kilobase (kb) regulatory region (marked by a red box) in a representative human gene screened in the creation of STaRRRT. As the length of the 5’-UTR can be markedly different among human genes, the 1 kb region downstream of the TSS will encompass the entire 5’-UTR for some but not all human genes. This is demonstrated by the marking of two possible start codons in relation to the regulatory region screened.

The resource, STaRRRT, was designed to identify tandem repeats in the regulatory region of genes as these may alter transcription due to their location. Further, the discovery of polymorphic regulatory region tandem repeats can serve as genetic markers linked to traits. There are many definitions of what constitutes a gene regulatory region. Typically, eukaryotic genes contain a core promoter, which is about 100 bp long and centered at the transcription start site (TSS; Figure 1), and a proximal promoter about 250 bp immediately upstream and downstream of the TSS [41]. For our analyses, we define the core and proximal promoter as having the coordinates (−60 to +40 bp) and (−250 to +250 bp) respectively, relative to the TSS. There is evidence to show that some human promoters have control elements in the region −1000 to −500 bp upstream of the TSS that can reduce gene expression [42]. Similarly, the 5’-UTR is known to have regulatory control elements that effect transcription [43,44]. The STaRRRT resource covers a 3 kb region spanning −2000 to +1000, with respect to the TSS (Figure 1). Further rationale for the selection of this region is given in the Methods.

To increase the utility of STaRRRT, the resource is restricted to short tandem repeats (STRs), due to their abundance, polymorphic nature and frequent use as genetic markers. In order to increase the chance of variable STRs being predominately represented in STaRRRT, we have restricted the purity to greater than or equal to 90%. We define an STR, also known as a microsatellite, as those TRs with period of 1 to 9 bp. Tandem repeats were identified from the UCSC ‘simpleRepeats’ table, which contains output from the Tandem Repeat Finder (TRF) program [39]. TRF uses distribution theory to detect TRs and also uses a minimum alignment score, with smaller period TRs requiring higher numbers of repeats to qualify. The ‘simpleRepeats’ table does not explicitly specify the TRF input parameters - minimum score, scoring weights, mismatch penalties, nor the matching probability (*P*_M_) or indel probability (*P*_I_). We determined some of these parameters empirically. Within the table the minimum reported score was found to be 50 and dividing this by the product of the period by the number of repeats shows the scoring weight must be set as 2. This infers the minimum reported STR size is 25 bp in length.

The STaRRRT resource is a spreadsheet that outlines the position and characteristics of 5,264 STRs present in a 3 kb regulatory region upstream of 4,448 human NCBI Reference Sequence gene transcripts (RefSeq, release 56 gene table; 43,284 total transcripts, 41,007 not in haplotypic regions or unplaced contigs) [45]. STaRRRT characterises each STR by giving, among other details: the position of the STR in relation to the transcription start site (TSS) of the gene (TxPos), the position of the STR in the genome (chromosome number and the strand on which it is situated), the period (length of the repeated unit), the number of repeats, the consensus sequence (or motif), and the purity of the repeat (being the percent match to the consensus sequence). A complete outline of the details provided in STaRRRT is shown in Table 1 and a sample of the STaRRRT resource is provided as Table 2. STaRRRT is publically available and can be accessed at http://www.newcastleinnovationhealth.com.au/STaRRRT. By using the various identifiers, genome locations or metrics, users can search, sort, filter or merge other data with STaRRRT without the need for extensive bioinformatics knowledge and experience. These tasks can be handled within Excel® (Microsoft® software) or by importing the table into a relational database.

**Table 1:** Details provided in STaRRRT

**Column name**	**Description of field**	**Example of entry**
Chrom	Chromosome number on which STR is located	chr1
chromStart	Start position on chromosome of the gene	28218048
chromEnd	End position on chromosome of the gene	28241236
cdsStart	Coding sequence start	28218673
cdsEnd	Coding sequence end	28240954
Strand	Strand on which the gene occurs	₋ (negative)
knownGeneId	KnownGene database identifier	uc001bpe.1
refSeqId^1^	RefSeq database identifier	NM_002946
ensGeneId	Ensembl database identifier	ENST00000373912
sourceAcc	GenBank transcript accession number	NM_002946.3
hgncSymbol^2^	HGNC gene symbol	RPA2
U133Id	Affymetrix GeneChip array identifier	U133A:201756_at;
U133Plus2Id	Affymetrix GeneChip Plus2.0 array identifier	201756_at
Category	Type of gene (coding or noncoding)	coding
txPos^3^	Position in relation to the TSS	−1910
srStart^4^	Start position on chromosome for the STR	28243107
srEnd	End position on chromosome for the STR	28243146
Period^5^	Length of the repeat unit in the STR	2
numRepeats	Number of copies of the repeat unit	19.5
srLength	Total length of the STR	39
consensusSize	Number of bases in the consensus sequence	2
perMatch^6^	% match of STR to consensus sequence; purity	100
perIndel	Percent insertions and/or deletions in the STR	0
Score	Alignment score (minimum = 50)	78
A	Percent of A's (adenine) in the repeat unit	0
C	Percent of C's (cytosine) in the repeat unit	0
G	Percent of G's (guanine) in the repeat unit	48
T	Percent of T's (thymine) in the repeat unit	51
Entropy	Entropy	1
Sequence	Consensus sequence of the repeat unit; motif	TG

^1^An STR only appears in STaRRRT if the gene has a RefSeq database identifier; ^2^An STR only appears in STaRRRT if the gene has an HGNC Gene Symbol; ^3^txPos was limited to −2000 to +1000 bp in the creation of STaRRRT; ^4^sr = simple repeats, as appears in the UCSC Genome Browser; ^5^Period was limited to 1 to 9 bp; ^6^perMatch was limited to ≥ 90%.

**Table 2:** Sample of the resource STaRRRT

**Chrom**	**Chrom start**	**Chrom end**	**Strand**	**refSeqId**	**hgncSymbol**	**Category**	**tx Pos**	**srStart**	**srEnd**	**Period**	**Num repeats**	**sr Length**	**per Match**	**A**	**C**	**G**	**T**	**Sequence**
chr1	1102483	1102578	+	NR_029639	MIR200B	noncoding	−586	1101897	1101928	6	5.2	31	92	19	80	0	0	CACCCC
chr1	1103242	1103332	+	NR_029834	MIR200A	noncoding	−1345	1101897	1101928	6	5.2	31	92	19	80	0	0	CACCCC
chr1	1631377	1633247	+	NR_002946	MMP23A	coding	−340	1631037	1631077	9	4.4	40	93	2	10	62	25	GTGTGCGGG
chr1	1950767	1962192	+	NM_000815	GABRD	coding	−994	1949773	1949836	5	12.8	63	98	61	17	0	20	ATAAC
chr1	2487804	2495188	+	NM_003820	TNFRSF14	coding	183	2487987	2488012	6	4.2	25	100	0	32	0	68	TTCTCT
chr1	2985741	3355185	+	NM_022114	PRDM16	coding	−121	2985620	2985645	3	8.3	25	100	0	32	68	0	GGC
chr1	3816967	3832011	+	NR_024455	LOC100133612	noncoding	−1887	3815080	3815118	3	12.7	38	100	68	31	0	0	AAC
chr1	6673755	6684093	+	NM_153812	PHF13	coding	−494	6673261	6673286	7	3.6	25	100	16	16	68	0	AGCGGGG
chr1	9352940	9429590	+	NM_025106	SPSB1	coding	−1705	9351235	9351260	1	25	25	100	0	0	0	100	T
chr1	9352940	9429590	+	NM_025106	SPSB1	coding	−157	9352783	9352812	7	4.1	29	100	0	72	27	0	CGCGCCC

This simplified sample of STaRRRT shows the details for the first 10 STRs in STaRRRT. The number of columns have been reduced from 30 to 19 shown here due to size limitations. STaRRRT can be viewed in its entirety at http://www.newcastleinnovationhealth.com.au/STaRRRT.

Downstream of the TSS, STaRRRT STRs may be located within the 5’-UTR or the coding region. We note 15,029 transcripts of the 41,007 (non-haplotype or unplaced contig) transcripts present in RefSeq (release 56 database) have 5’-UTR regions that will go beyond the 1 kb downstream limit of this resource (Figure 1); hence, STaRRRT is not comprehensive for all STRs in 5’-UTRs. Similarly, for the 25,978 transcripts with a 5’-UTR shorter than 1 kb, an STR (or STRs) presented in STaRRRT may be present in the coding region. The position of the STR within the upstream region, 5’-UTR or coding region can be calculated by comparing the srStart:srEnd coordinates with the chromStart:chromEnd (transcription start and end) and cdsStart:cdsEnd (coding sequence start and end) coordinates.

### General characteristics of STaRRRT STRs relative to genic or all STRs

Of the 41,007 (non-haplotype or unplaced contig) transcripts present in RefSeq (release 56 database), 4,448 gene transcripts (within 4,441 unique gene loci) were found to contain at least one STR with purity of at least 90% in the 3 kb regulatory region analysed (Figure 1); so, 18.8% of all genes in the human genome.

**Figure 2:**
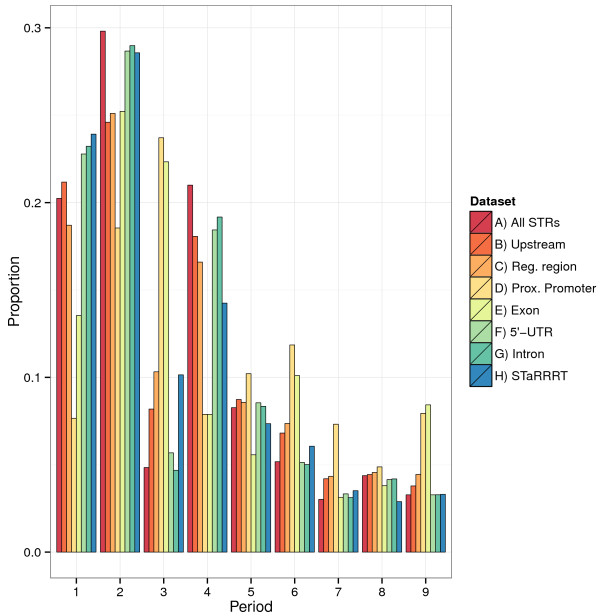
**Comparison of STRs of different period lengths in the whole human genome, gene coding regions and STaRRRT STRs.** This histogram shows the proportion of STRs present in STaRRRT having different period (“STaRRRT”) compared to the proportions across the whole human genome (“All STRs”), in the 2 kb upstream region (−2000, -1; “Upstream”), in the 3 kb region analysed for all STRs (with no purity restriction, “Reg. region”), in the proximal promoter (−250, +250; “Prox. Promoter”), in exons (“Exon”), in 5’-UTRs (“5’-UTR”), and in introns (“Intron”).

The most common STRs throughout the human genome are dinucleotides, and this is also the case for STaRRRT STRs (Figure 2). Together, STRs with periods of 1 and 2 comprise over half of all STRs in the STaRRRT resource, with frequencies of 23.9% and 28.6%, respectively. Compared with all (genome-wide) STRs, there are a higher proportion of STRs with period of 1, 3, 6, 7 and 9 in the STaRRRT resource and less with periods of 4 or 8 (Figure 2). For STRs in STaRRRT, the number of STRs generally decreases as the period increases (exceptions include period 2, 4 and 9). This is a similar distribution to that observed for tandem repeats in noncoding regions of the human genome [4]. As STaRRRT STRs may overlap the upstream region (−2000, -1 bp), proximal promoter (−250, +250 bp), 5’-UTR and exons, the distribution of STaRRRT STRs by period has features observed in each of these separate distributions (Figure 2). Comparison of STaRRRT STRs to the unfiltered set of STRs in the regulatory region shows the 90% purity filter of STaRRRT increases the proportion of period 1 and 2 STRs markedly, while STRs from all other periods are reduced. The period 1 and 2 frequencies observed in STaRRRT more closely resemble that of 5’-UTRs and introns (Figure 2).

We note the more than 2-fold increase in the frequency of STaRRRT STRs (relative to all STRs) with period of 3. This is likely due to the encompassing of the proximal promoter in the regulatory region and the inclusion of some exon regions downstream of the TSS. Compared to all categories other than exons, the number of period 3 STRs in proximal promoters is more than 4-fold increased. More broadly, the distribution of STRs in proximal promoters with a multiple of 3 (being period 3, 6 and 9) is very similar to that in exons (Figure 2). This increase is offset by the relative decrease in frequency of STRs with period 1 and 2.

### Distribution of STaRRRT STRs show distinct trends at the TSS and in the proximal and core promoters

**Figure 3:**
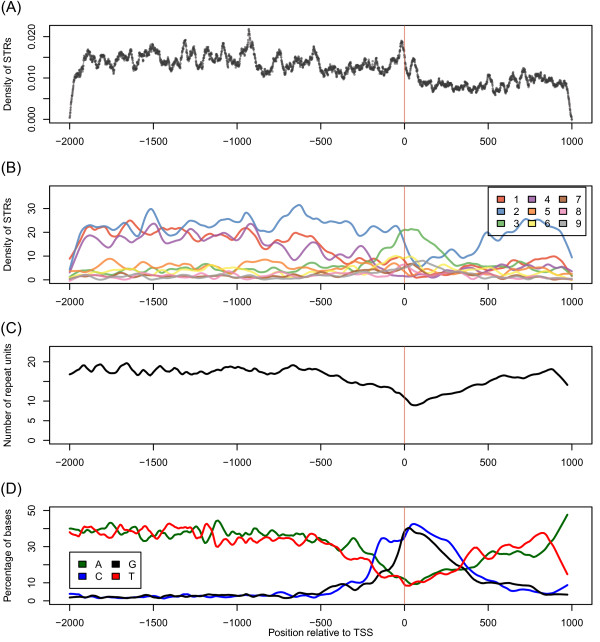
**Summary plots across the TSS.** The distribution of STRs in the upstream regulatory region of the human genome shows distinct trends around the TSS and core promoter. All lines are smoothed by LOWESS (locally weighted scatterplot smoothing) regression. **(A)** The density of STaRRRT STRs across the 3 kb upstream regulatory region. This run chart shows the STR density of the 5,264 STRs from STaRRRT at each base position in the regulatory region with a regression line also fitted to the data. **(B)** STaRRRT STR density decomposed into periods. **(C)** The number of STR repeat units across the TSS. **(D)** The percentage of bases in each STR across the TSS.

To assess the nature of the (high purity) STR distribution over the 3 kb regulatory region, STaRRRT period, base composition and repeat unit length distributions were plotted with respect to the TSS coordinate (TxPos). Examination of the density of STRs relative to the TSS shows a non-uniform distribution with more STaRRRT STRs upstream of the TSS (Figure 3A) and characteristics of a wave-like unevenness in density. Downstream of the TSS, the overall density of STRs is reduced and the local peaks and troughs in density are less distinct.

When the repeats in Figure 3A are decomposed into subpopulations classified by repeat period, a number of trends emerge (Figure 3B). The most striking observation is the increased density of repeats with period of 3 (trinucleotides; shown in green) in the region approximately 300 bases upstream and downstream of the TSS and the predominance of repeats with period of 2 (dinucleotides; shown in blue) in the region +300 to +1000, downstream of the TSS. Upstream of the TSS, peaks and troughs in repeat density are present with some regularity; in particular, the density of STRs with periods of 2, 4 and 5. Using waves as an analogy, in the region −2000 bases to approximately −800 bases, relative to the TSS, the densities of STRs with periods of 2 and 4 are in phase before becoming anti-phased from −800 bases until approximately −200 bases, relative to the TSS. This change in phase coincides with an increase in the abundance of STRs with period of 5.

The base composition and repeat unit length of STRs in the regulatory region also have distinct patterns. For the most part, repeats are AT-rich; however, there is a profound change towards GC-rich repeats, with fewer repeat units surrounding the TSS (Figure 3C and D). This region of change correlates strongly with the large increase in period 3 (trinucleotide) repeats noted earlier (Figure 3B). These GC-rich, relatively low repeat unit trinucleotide repeats overlap with the proximal promoter, defined as 250 upstream to 250 downstream of the TSS (−250, +250; [41] and more specifically with the core promoter, which we define here as 60 bp upstream to 40 bp downstream of the TSS (−60, +40). A further decomposition of the data in Figure 3 into 3,479 CpG island overlapping and 1,785 non-CpG island overlapping regulatory regions shows the TSS proximal GC-rich, trinucleotide repeats are situated particularly in CpG island containing regulatory regions (Additional file 1: Figure S1). Interestingly, the smaller set of regulatory regions without an overlapping CpG island, seem to exclude STRs in the region just before the TSS until approximately 100 bp downstream (Additional file 1: Figure S2). They also exhibit a periodic and anti-phased increase and decrease in adenine and thymine base composition.

### STaRRRT STRs are found in genes involved in metabolism, signal transduction and the neurological system

To determine if STRs are associated with particular biological pathways or processes, the 4,441 gene loci within STaRRRT were analysed with gene set enrichment analysis (GSEA) methods; the controlled vocabulary approach implemented in the H-Invitational Database (H-InvDB) Enrichment Analysis Tool (HEAT; http://h-invitational.jp/hinv/ahg-db/index.jsp; [46] and the expert curation and literature mining approach in the Ingenuity® Pathways Analysis software (IPA; Ingenuity® Systems, http://www.ingenuity.com). Two disparate GSEA methods were used for comparison and we gave more weight to the interpretation of findings consistent to both GSEA approaches. As the degree of STR polymorphism in intragenic regions is also high [15], we considered the possibility that the enrichment we observed is not limited to the regulatory region, but is instead representative of a broader genic enrichment signature. For the HEAT analysis, we compared the STaRRRT gene set enrichment findings to those of genes with STRs in the intragenic region, so exons and introns. In the STaRRRT set, the 5,264 STRs across the 4,441 gene loci were mapped to 3,258 H-InvDB transcript (HIT) identifiers (IDs) and analysed using the HEAT web tool. For the exonic STR gene set we gathered all genes with at least one STR in any exon regardless of purity (3,287 STRs in 2,617 genes, mapping to 2,228 HIT IDs). As the intronic region is typically much larger than the exonic or regulatory region we found approximately a third of all genes (13,361 genes, 24972 HIT IDs) had a least one STR in an intron. We reduced these genes down to a size more appropriate for gene set enrichment analysis and comparable to that in the STaRRRT and exonic sets. This reduction was performed using two approaches; a filtering and a random subset approach. Filtering was performed by limiting analyses to those genes with the highest quartile of ≥ 90% purity STRs per kilobase of intron. This intentional bias was based on the assumption that genes with the uppermost high purity intronic STR densities are more likely to have polymorphic STRs under evolutionary selection. Filtering created a set of 17,482 STRs in 3,444 genes mapping to 2,795 HIT IDs in total. For the random subsets, ten random samples of genes containing ≥ 90% purity intronic STRs were subjected to HEAT analysis. Each set had the same number of HIT IDs as the STaRRRT STR gene set (3,258). We found some degree of variance in the number of significant terms, particularly for KEGG pathways (Additional file 1: Table S2). However, the means of the number of significant terms were similar to the high density set (Additional file 1: Table S2). Given this variance, we only report significant terms where the majority of samples (at least 6 from 10 samples) agreed. These results are presented in Additional file 1: Table S3. In comparing the two intron methods, the filtering method reported 21 KEGG pathways as enriched (FDR p < 0.05; Table 3), while the random subset method found 6 pathways enriched (Additional file 1: Table S3). The high density intron set intersected to a high degree with STaRRRT (15 from 21 pathways) and the random sample intron set (5 from 6 pathways), so we concentrated on this set in later analyses.

**Table 3:** KEGG pathway results from HEAT analysis grouped by pathway class

			**STaRRRT**		**Exon**		**Intron**	
**Term**	**ID**	**Genes**	**Enrich**	**p-value**	**Enrich**	**p-value**	**Enrich**	**p-value**
**Metabolism**								
Purine metabolism	230	126	1.94	0.020	-	-	2.44	0.006
Glycine, serine and threonine metabolism	260	53	-	-	-	-	3.33	0.006
Glycosaminoglycan degradation	531	12	-	-	7.14	0.006	-	-
Inositol phosphate metabolism	562	54	3.04	0.002	-	-	2.70	0.028
Glycan structures - biosynthesis 1	1030	40	3.24	0.005	-	-	-	-
Glycan structures - degradation	1032	18	-	-	6.00	0.006	-	-
**Development/Cell growth and death**								
Apoptosis	4210	111	2.21	0.006	2.13	0.050	2.11	0.028
Dorso-ventral axis formation	4320	80	2.03	0.048	-	-	2.73	0.007
Axon guidance	4360	114	2.25	0.004	2.38	0.016	2.18	0.020
**Signal transduction/Environmental information processing/Cell communication/Cell motility**								
Calcium signaling pathway	4020	108	3.23	8.6E-07	2.50	0.012	2.30	0.015
Phosphatidylinositol signaling system	4070	64	3.09	0.001	2.57	0.050	2.50	0.028
Wnt signaling pathway	4310	126	2.32	0.002	2.14	0.029	-	-
VEGF signaling pathway	4370	155	2.33	0.001	2.09	0.020	1.89	0.032
Focal adhesion	4510	120	2.33	0.002	2.42	0.012	2.20	0.015
Adherens junction	4520	166	1.76	0.031	2.50	0.002	2.30	0.004
Tight junction	4530	101	1.95	0.038	2.68	0.007	-	-
Gap junction	4540	116	2.32	0.002	2.19	0.032	2.41	0.007
Jak-STAT signaling pathway	4630	140	-	-	2.73	0.002	-	-
Regulation of actin cytoskeleton	4810	98	2.26	0.007	2.41	0.024	2.24	0.026
**Immune system**								
Hematopoietic cell lineage	4640	19	-	-	-	-	5.39	0.006
T cell receptor signaling pathway	4660	167	1.89	0.011	2.50	0.002	-	-
B cell receptor signaling pathway	4662	160	1.75	0.037	2.61	0.002	-	-
Leukocyte transendothelial migration	4670	99	1.88	0.051	2.73	0.006	2.06	0.043
**Nervous system**								
Long-term potentiation	4720	125	2.34	0.002	2.17	0.028	-	-
Long-term depression	4730	142	2.21	0.002	2.18	0.020	1.96	0.028
**Endocrine system**								
Insulin signaling pathway	4910	195	1.98	0.002	2.32	0.002	2.18	0.004
Adipocytokine signaling pathway	4920	150	-	-	3.01	1.3E-04	1.96	0.028
**Human diseases**								
Type II diabetes mellitus	4930	22	3.16	0.051	-	-	5.33	0.004
Epithelial cell sig. in *H. pylori* infection	5120	150	2.17	0.002	2.41	0.006	2.16	0.010
Colorectal cancer	5210	82	2.00	0.051	-	-	2.68	0.008

Results from gene set enrichment analysis of the set of transcripts with STaRRRT STRs are shown alongside results for transcripts with STRs located in exons and transcripts with the highest density of high purity STRs in the introns. Results shown are FDR-corrected p-values. A KEGG pathway is only presented in the table if at least one of the STaRRRT, exon or high density intron results has an FDR-corrected p-value of less than 0.01. Columns with “-“ characters are those sets unenriched (so p > 0.05 before FDR correction). “Genes” is the number of entities in each set and “Enrich” is the ratio of the number of transcripts observed with STRs relative to that expected.

The KEGG pathways highlighted by the HEAT analysis clustered around particular cell functions. The pathways, grouped by KEGG Cellular Process, were associated with nucleotide, amino acid and carbohydrate metabolism, development, cell growth and death, signal transduction, environmental information processing, cellular communication and motility, and the immune, nervous and endocrine systems (Table 3). Interestingly, the KEGG gene set enrichment analyses of STaRRRT, exonic and the high density-enriched intronic STRs produced very similar results; of the 21 STaRRRT enriched pathways (FDR p < 0.05), 15 pathways were similarly enriched (FDR p < 0.05) in the exonic and high-density intronic sets analysed (Table 3) and all analyses identified a strong enrichment for expression in neural tissue (Table 4), with STaRRRT genes showing a particularly strong enrichment (p = 4.0 × 10^-10^). The differences between STaRRRT and intragenic STR genes were mostly in pathways associated with carbohydrate metabolism, calcium and adipocytokine signaling. The calcium signaling pathway is the most enriched KEGG pathway for STaRRRT STRs (p = 8.56 × 10^-7^) but is considerably less enriched for exonic (p = 0.0117) and high-density intronic STR genes (p = 0.0145). We also note, in contrast to intragenic STR genes, STaRRRT genes are particularly expressed in the skeletal/cardiac muscle tissue (Table 4) and are more abundantly located in the endoplasmic reticulum and plasma membrane of the cell (GO Cellular Components, Additional file 1: Table S1). Also, STaRRRT STR genes are associated with a larger number and hence wider range of biological processes and molecular functions than intragenic STR genes (GO Biological Process, GO Molecular Function; Additional file 1: Table S1).

**Table 4:** Tissue-specific expression results from HEAT analysis

		**STaRRRT**		**Exon**		**Intron**	
**Tissue**	**Genes**	**Enrich**	**p-value**	**Enrich**	**p-value**	**Enrich**	**p-value**
Kidney/bladder	139	-	-	2.08	0.014	2.21	0.003
Muscle/heart	168	2.01	0.001	-	-	-	-
Neural	393	2.23	4.0E-10	1.80	0.003	2.01	8.1E-06
Placenta/testis/ovary	198	1.88	0.001	1.93	0.014	-	-

A description of the columns is given in Table 3.

The IPA Top Canonical Pathways and Top Bio Functions analyses (Table 5) were in strong agreement with the HEAT results. Again, most pathways were associated with signal transduction, metabolism, cell growth and death and immune, endocrine and nervous system function. Interestingly, in the IPA Diseases and Disorders analysis, several have a neurological basis; with neurological disease (including mood disorders (p = 1.81 × 10^-4^), Huntington’s disease (p = 0.00571), neuromuscular disease (p = 0.00878) and major depression (p = 0.0173)) and psychological disorders (including schizophrenia (p = 0.00289), bipolar disorder (p = 4.06 × 10^-4^) and depressive disorder (p = 0.00286)) listed as the top two (Table 5).

**Table 5:** IPA results

**Top Bio Functions**	**Molecules (n)**	**p-value**
**Diseases and disorders**		
Neurological disease	443	1.27E-04 - 4.94E-02
Psychological disorders	236	1.81E-04 - 4.94E-02
Developmental disorder	132	9.19E-04 - 4.24E-02
Antimicrobial response	29	1.38E-03 - 2.00E-02
Infectious disease	418	2.25E-03 - 4.17E-02
**Molecular and cellular functions**		
Cellular movement	325	3.39E-04 - 4.81E-02
Cell death and survival	501	6.18E-04 - 4.83E-02
Cell-to-cell signaling and interaction	119	1.07E-03 - 4.81E-02
Cellular development	290	1.17E-03 - 4.37E-02
Cellular growth and proliferation	192	1.47E-03 - 4.81E-02
**Physiological system development and functions**		
Cardiovascular system development and function	167	7.56E-06 - 4.70E-02
Organismal development	146	3.20E-05 - 4.37E-02
Humoral immune response	12	1.38E-03 - 4.81E-02
Reproductive system development and function	31	1.47E-03 - 4.17E-02
Hematological system development and function	107	1.74E-03 - 4.81E-02
**Top 20 canonical pathways**	**Ratio**	**p-value**
NGF signaling	34/111 (0.306)	3.16E-03
Pyridoxal 5'-phosphate salvage pathway	22/62 (0.355)	4.22E-03
Reelin signaling in neurons	26/82 (0.317)	6.29E-03
Neuropathic pain signaling in dorsal horn neurons	31/102 (0.304)	6.92E-03
GNRH signaling	38/135 (0.281)	7.00E-03
Cellular effects of sildenafil (Viagra)	37/127 (0.291)	9.28E-03
Calcium signaling	48/189 (0.254)	1.01E-02
Factors promoting cardiogenesis in vertebrates	27/91 (0.297)	1.27E-02
Synaptic long-term depression	39/142 (0.275)	1.51E-02
B cell receptor signaling	43/162 (0.265)	1.95E-02
FGF signaling	26/88 (0.295)	2.01E-02
mTOR signaling	49/189 (0.259)	2.06E-02
Gɑq signaling	40/157 (0.255)	2.33E-02
Dopamine-DARPP32 feedback in cAMP signaling	43/161 (0.267)	2.40E-02
D-myo-inositol (1,4,5)-triphosphate biosynthesis	10/26 (0.385)	2.66E-02
PPARɑ/RXRɑ activation	44/173 (0.254)	2.86E-02
NF-κB activation by viruses	22/79 (0.278)	3.18E-02
Xenobiotic metabolism signaling	66/268 (0.246)	3.20E-02
Antioxidant action of vitamin C	27/98 (0.276)	3.43E-02
Maturity onset diabetes of young (MODY) signaling	8/22 (0.364)	3.64E-02

Results from comparison of the set of transcripts containing STaRRRT STRs with the reference set Ingenuity Knowledge Base are shown. For “Top Bio Functions”, the number of molecules (n) relates to genes containing STaRRRT STRs in each enriched functional group. For “Top Canonical Pathways”, the number of STR-containing genes, relative to the total number of genes for each canonical pathway, is shown as a fraction and as a ratio (in brackets). Results shown are limited to those with a p-value less than 0.05 for the “Top Bio Functions” and the 20 most significant results with a p-value less than 0.05 for the “Top Canonical Pathways”.

Collectively, the GSEA results show that genes with STRs in the regulatory region or exons, or those genes with high intronic STR density, have enrichments for largely the same classes of gene pathways. These pathways are primarily associated with metabolism, signal transduction, environmental information processing, development, cell growth, death, motility and communication and immune, nervous and endocrine system function. There are some differences between the STaRRRT, exonic and high-density intronic gene sets in KEGG pathways. Broadly, STaRRRT genes have more numerous enrichments and are particularly enriched for calcium signaling.

## Discussion

By genome-wide analysis, this study has identified that 18.8% of all human genes contain at least one highly pure STR in their upstream regulatory region. This is consistent with the previous suggestion that TRs of all period lengths are present within promoter regions of 10 to 20% of human genes [4]. The upstream promoter region appears to consist of predominantly short (mostly with repeat period of 1 and 2), AT-rich sequences, which is concordant with the findings of Vinces *et al.*[5] in the yeast genome and Sawaya *et al.*[2] in human promoters. We demonstrate that in humans, the proximal promoter (−250, +250) and in particular the region overlapping the typical core promoter region (−60, +40) have GC-rich STRs. As approximately 72% of human promoters have high GC-content [47,48] with CpG island density reaching a maximum near the TSS [47], we reason this increase in STR GC-content reflects the underlying GC-rich promoter sequence.

Consistent with a previous genome-wide survey of all STRs [1], period 2 STRs (dinucleotides) are the most abundant STRs in the regulatory region across human genes. Likewise, the distribution of STaRRRT STRs across repeat periods is very similar to that reported by Gemayel *et al.* (2010) for the distribution of all TRs in noncoding regions across the human genome [4]. However, similar to coding regions, we find a striking enrichment of trinucleotide repeats (period 3 STRs) in the proximal promoter region, both upstream and downstream of the TSS (Figure 3B). The similarity of this enrichment signature in regulatory regions to that observed in coding regions [3] is a significant and novel finding, and adds weight to the likely functional significance of these results.

STRs in coding regions almost exclusively have a repeat period which is a multiple of 3 bases [4]; this is thought to be due to the nature of triplet codons and selection against frameshift mutations [49]. While the region upstream of the TSS is not transcribed, the abundance of trinucleotide repeats suggests a selection pressure of similar magnitude to that observed in coding regions [3,50]. Possible explanations include alternative translation start sites or other functional constraints, possibly related to chromatin structure, nucleosome positioning and/or transcription factor activity. We note that high abundance TSS proximal GC-rich repeats and trinucleoide repeats are only associated with regulatory regions overlapping CpG islands. Interestingly, the smaller non-CpG island overlapping group is composed of mostly dinucleotides repeats and in the region approximately −500 to 500 bp around the TSS the repeats have a regular wavelike increase and decrease in adenine and thymine abundance. We speculate this pattern may be associated with nucleosome positioning.

Broadly, we suggest that the distribution of STRs around the promoter has functional significance, as also proposed recently by Sawaya *et al.*[2] following their discovery of a high density of STRs at the TSS and by Kozlowski *et al.*[3] who found non-random distribution of trinucleotide repeats in the exome. Altered TR length in or near core promoters can change local nucleosome positioning, is likely to hinder transcription factor binding and therefore affect rates of transcription and hence gene expression [51,52]. It has been shown that changes as small as 2 bp in nucleosome positioning can alter promoter activity [52]. Moreover, it has been shown in yeast that nucleosome position is negatively correlated with the positioning of TRs [5]. Hence, our findings of profound changes in STR period, repeat unit number and base composition around the TSS of human genes is interesting given the findings in yeast and indicate that similar mechanisms of regulating gene expression may be at play in the human genome [52]. In this regard, a recent study has shown that a polymorphic GA-repeat in the human *SOX5* gene promoter can affect gene expression, with the longer allele resulting in a 2.7-fold increase in activity [53]. The authors report this as first evidence of a functional STR in a human gene core promoter [53].

Controlled vocabulary gene set enrichment analysis of gene transcripts with STaRRRT STRs in the regulatory region found a number of significantly enriched KEGG pathways, GO terms and tissues enriched for expression of these genes. These findings have broad overlap with gene set enrichment of gene transcripts having STRs in the exons and those gene transcripts with a high density of STRs in the intronic regions. Regulatory region, exon and intron analyses all show enrichment for expression in neural tissue. Enrichment of neurological genes and pathways in the STaRRRT analysis is consistent with the known role of TRs in neurodegenerative and neurodevelopmental disorders [37]. Several neurological diseases known to be caused by variable TRs also appeared in the STaRRRT IPA results, namely Huntington’s disease and neuromuscular disease, as well as major depression which has a known association with a variable TR [54]. STaRRRT can be used to analyse the role STRs may play in the development of various diseases, such as neurological disorders and cancer in which they have already been implicated. This could potentially lead to the identification of targets for diagnosing and treating diseases.

While the STaRRRT, exonic and intronic gene set enrichment results show a very high degree of overlap, we also note some differences between the enrichment signatures. The calcium signaling pathway was the most enriched KEGG pathway for STaRRRT STRs but is only mildly enriched in the exonic and intronic gene sets. In particular, STRs were significantly enriched in the regulatory region of genes involved in the calcium signaling pathway (KEGG), calcium ion binding (GO Molecular Function) and ion transport and activity (GO Biological Process and Function, respectively, which includes calcium transporters). Intracellular calcium signaling regulates a plethora of cellular processes including apoptosis, gene transcription, proliferation, cell cycle progression and differentiation [55]. Disruption is associated with a number of diseases such as Alzheimer’s disease, diabetes, skin disorders, cardiac disease and cancer [56]. Previous studies have shown STRs can impact calcium signaling with the identification of an expansion in the CAG repeat in exon 1 of isoforms ‘a’ and ‘c’ of *KCNN3* and the 5’-UTR of isoform ‘b’ of *KCNN3*, which encodes a calcium activated potassium channel [45,57]. The expanded variant of *KCNN3* has been reported to reduce channel conductance and is associated with better cognitive performance of individuals with schizophrenia [57]. An enriched presence of STRs in the regulatory region of the calcium signaling machinery has not previously been reported and may have significant consequences for protein expression and function and consequently disease. Further, the second most enriched KEGG pathway, vascular endothelial growth factor (VEGF) signaling, is associated with vasculogenesis and angiogenesis. We note that only STaRRRT genes were enriched for expression in skeletal and cardiac muscle and in the IPA analysis, cardiovascular system development and function was listed as the most enriched physiological system (Table 5).

The GSEA findings are consistent with mechanisms of human evolution. Due to their inherent instability, the presence of variable STRs in regulatory regions may act as a flexible switch to allow ready adaptation through positive selection with implications for human evolution and disease. The enrichment of neural processes and pathways is concordant with the involvement of TRs in the evolution of cognition and behaviour [58], supporting the idea of Legendre *et al.* (2007) that repeats may play a role in the swift evolution of the primate brain. The over-representation of STaRRRT genes involved in transcriptional regulation (Additional file 1: Table S1) further supports a role for STRs in evolutionary mechanisms, given the suggested role for polymorphic TRs in modifying transcription and leading to rapid evolutionary changes [59,60]. Haygood et al. (2007) surveyed base substitution rates in human genomic regions upstream of the TSS and compared these with neighbouring intronic sequence and also substitution rates in chimpanzees. High rates of base substitution (compared to intronic rates) in human, but not chimpanzee promoters, were observed in genes involved in neuronal function, development, glycolysis and carbohydrate metabolism, protein folding, vision, oncogenesis and anion transport [61]. This list of enriched biological processes shows much resemblance with the current study. Therefore, we hypothesise that the set of enriched STaRRRT STRs is reflective of general positive selection in human promoter regions since our divergence from chimpanzees.

The importance of STRs has been recognised due to their abundance in the human genome, high mutation rates, and relevance to disease phenotypes and evolutionary processes. As technologies improve and analysis of repetitive sequences becomes simpler and more cost effective, resources such as STaRRRT will become more valuable and commonly utilised in biological studies. Further applications for the use of STRs include the study of how environmental factors (such as radiation or toxic compounds) affect genomic mutation rate [7], which would rely upon a thorough understanding of the baseline mutation rates and other characteristics of STRs in the human genome.

## Conclusions

STaRRRT acts as a starting point for researchers interested in looking at the role of STRs in promoter regions throughout the human genome. It is publically available and can be accessed at http://www.newcastleinnovationhealth.com.au/STaRRRT. This resource is suitable for researchers with limited bioinformatics experience who are interested in specific STRs, genes or phenotypes. Multiple database identifiers are available in STaRRRT including Affymetrix array probeset identifiers which allow legacy gene expression data to be easily mapped to this table.

This paper presents the findings from investigations of the distribution and abundance of STRs in the 5’ regulatory region of human genes. We have identified a unique signature of STR enrichment in this regulatory region which is most pronounced within neural genes, and calcium signaling and neurological pathways. This functional signature of STR enrichment in the regulatory regions of genes is similar to that previously identified in coding regions, suggesting that regulatory region STRs are subject to similar evolutionary pressures and may have an important role in gene expression. Hence, this study has identified STRs likely to be involved in the expression of genes associated with particular disease phenotypes and recent evolution of the human genome.

## Methods

### Resource construction

The STaRRRT resource was constructed in a series of nested table joins in MySQL database (SQL commands provided in Additional file 2). The tables, in hg19 build coordinates, were downloaded from the UCSC Genome Browser (http://genome.ucsc.edu/index.html). The genome-wide table of tandem repeats identified by the Tandem Repeat Finder program [39] was reduced to the set of highly pure STRs by filtering for TRs with a length less than or equal to 9 bp and repeat purity of at least 90%. The analysis was then further restricted to those STRs proximal to the transcription start site (TSS) of genic loci with a RefSeq identifier. In instances where genic loci had more than one RefSeq transcript, the canonical transcript as defined by UCSC was used. For each canonical TSS, we entrained analyses to a span around the TSS rather than include all the 5’-UTR. This is due to approximately 11% of RefGene curated transcripts, in particular transcribed pseudogenes and noncoding genes, not having a defined 5’-UTR. The 5’-UTR is also highly variable in size; while most genes have a short 5’-UTR (median length of 292 bp and mean of 9885 bp), some genes have particularly long 5’-UTRs, for example, the transcript (NM_002839) of *PTPRD* has a 5’-UTR length of around 1.88 Mb.

For each STR in STaRRRT, containment within the regulatory region was defined as having start and end sites contained within the region 2 kb upstream of a TSS to 1 kb downstream (Figure 1). These STRs were given a relative coordinate with respect to the TSS (TxPos), defined as the number of nucleotides upstream or downstream from the STR start coordinate to the TSS. We also joined other identifiers (IDs) to this table such as KnownGene and Ensemble database IDs, NCBI RefSeq and GenBank accession numbers, HGNC gene symbols and Affymetrix array probeset IDs so legacy gene expression data can easily be mapped to this table. The final Short Tandem Repeats in Regulatory Regions table (STaRRRT) is a list of all the highly pure STRs present in the 3 kb regulatory region at the 5’-end of all human genes. This table was exported from MySQL into R (v2.15.0) and converted into an Excel spreadsheet. The SQL code used to construct the table is provided in Additional file 2.

### Analysis of density of STRs and base composition in relation to the TSS

Using the functionality of the ‘GenomicRanges’ R library, we calculated from all STRs in the genome the subsets that are located within exons, introns or 5’-UTRs and those STRs located upstream (−2,000 to −1 bp), in the proximal promoter (−250 to +250 bp) or regulatory region (−2,000 to +1,000 bp), relative to the TSS. An STR qualified as being located within an entity if some portion of it overlapped.

To calculate STR density, for each STR the start and end coordinates (relative to the TSS) were used to generate a sum of STRs at each base position across the regulatory region. The sums were used to form a density per base and these densities smoothed using LOWESS local regression. Similarly, the base composition and repeat unit lengths were calculated for each base position across the regulatory region and were smoothed using local regression. For further detail consult the R scripts or the HTML-based report in Additional file 2.

### Gene set enrichment analysis

Two gene set enrichment analysis approaches, the H-InvDB Enrichment Analysis Tool (HEAT; http://h-invitational.jp/HEAT/search.do) and Ingenuity Pathway Analysis (IPA; Ingenuity® Systems; http://www.ingenuity.com) were used to functionally characterise the list of genes from STaRRRT. For the HEAT analysis, KnownGene IDs within STaRRRT were mapped to HIT IDs (identifiers of an RNA transcript from the H-InvDB database), using the UCSC ‘knownToHInv’ table. Additional STR tables were prepared by filtering all STRs in the genome to those within exons and introns. Given the high number of transcripts with at least one STR in an intron we needed to reduce this set for GSEA. We created two sets; transcripts with a high-density of STRs in introns and randomly sampled transcripts with STRs in the introns. For the high-density set, filtering was introduced by limiting to those STRs with a purity ≥ 90% and to those genes with the highest quartile of STR density within the intronic region (one high purity STR per 7.32 kb intron). The density was calculated by summing the total intron width per gene and dividing this by the total number of STRs present in the introns of that gene. For the random sampling approach, ten HIT ID sets, each the same size as the STaRRRT set (3,258) were sampled from the 9,299 HIT IDs in the complete high purity intron set.

All sets were subjected to HEAT analysis and the returned tables were imported into R, processed and the p-values multiplicity corrected using a false discovery rate (FDR) correction from the Bioconductor ‘multtest’ library based upon the number of tests performed. An R script in Additional file 2 discloses all the processing steps.

For the IPA analysis, the list of 4,448 RefSeq gene transcript IDs was uploaded and, when compared against the reference set Ingenuity Knowledge Base (Genes Only), a list of 4,377 “analysis-ready molecules across observations” was created. A Core Analysis was run and the output included enrichment in the categories “Top Bio Functions” (including Diseases and Disorders, Molecular and Cellular Functions, and Physiological System Development and Function) and “Top Canonical Pathways”.

## Abbreviations

bp: Base pairs; FDR: False discovery rate; GO: Gene ontology; GSEA: Gene set enrichment analysis; GWAS: Genome-wide analysis study; HEAT: H-InvDB enrichment analysis tool; HIT: H-InvDB transcript; IDs: Identifiers; IPA: Ingenuity pathways analysis; kb: Kilobase; KCNN3: Potassium intermediate/small conductance calcium-activated channel, subfamily N, member 3 gene; KEGG: Kyoto encyclopedia of genes and genomes; RefSeq: NCBI reference sequence; SNP: Single nucleotide polymorphism; SQL: Structured query language; SSR: Simple sequence repeat; STR: Short tandem repeat; STaRRRT: Short tandem repeats in regulatory region table; TR: Tandem repeat; TSS: Transcription start site; TxPos: TSS coordinate; UCSC: University of California, Santa Cruz; UTR: Untranslated region; VEGF: Vascular endothelial growth factor; VNTR: Variable number of tandem repeats.

## Competing interests

The authors declare that they have no competing interests.

## Authors’ contributions

RJS conceived this study. All authors were involved in the study design and resource definition. KAB coordinated the study and managed the development of the manuscript. JPR constructed the resource and additional gene sets for comparison, and performed analysis of STR distribution. KAB and JPR performed the GSEA and wrote the paper. KAAK, DMG and NAB were involved in developing the manuscript. All authors read and approved the final manuscript.

## Supplementary Material

Additional file 1 Supplementary materials (Figures S1-S2; Tables S1-S3). Click here for file

Additional file 2 **Supplementary methods.** (SQL code; R scripts; R/Markdown HTML-based report). Click here for file
