# Supplementary figures and images for: STaRRRT: a table of short tandem repeats in regulatory regions of the human genome
Source: BMC Genomics. 2013 Nov 15;14:795. doi: 10.1186/1471-2164-14-795 (PMC3840602; doi:10.1186/1471-2164-14-795)

(A)

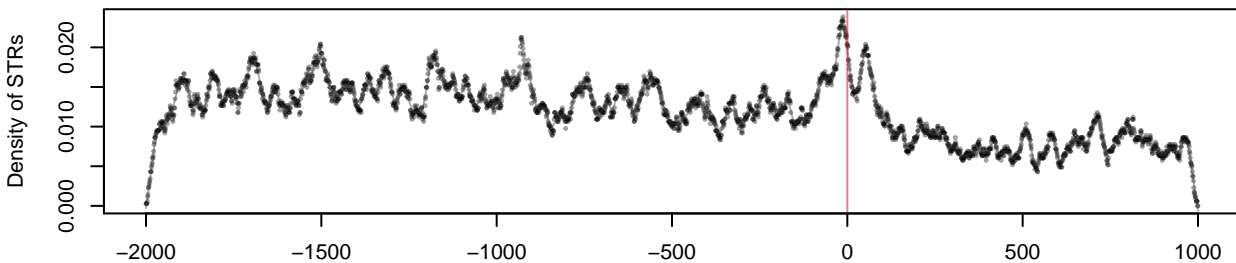

(B)

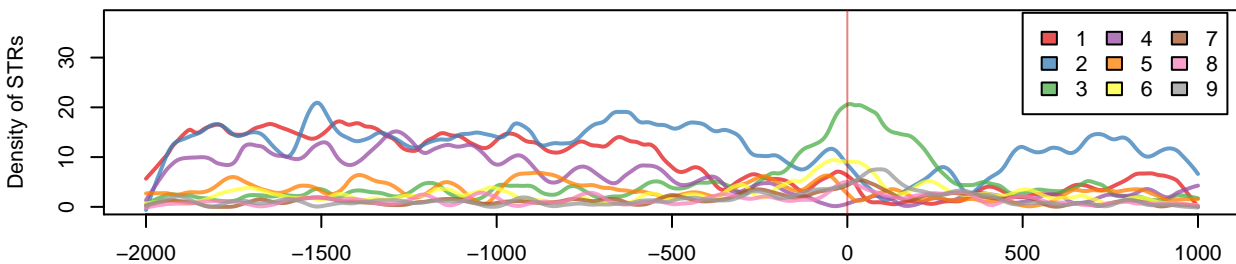

(C)

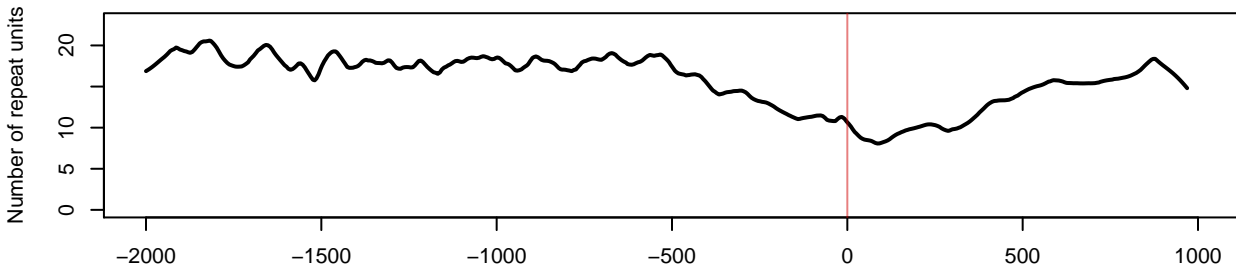

(D)

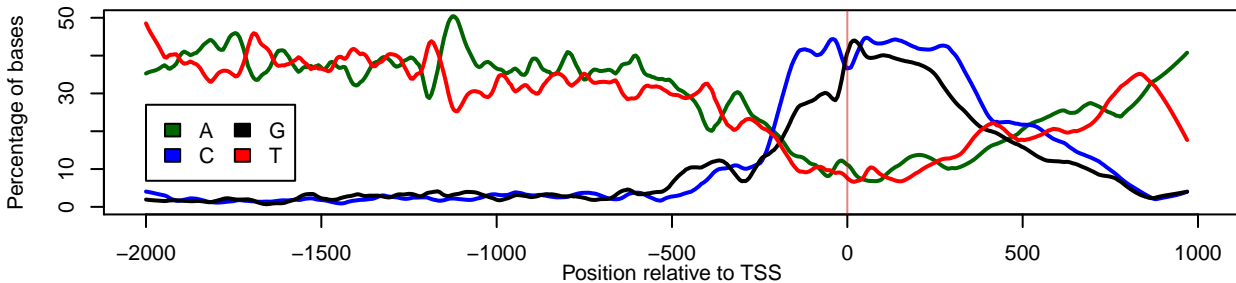

Supplement: Additional file 1 — Supplementary materials (Figures S1-S2; Tables S1-S3). [file 1471-2164-14-795-S1.zip › 1088845327100107_add1/Supp Fig1_(CpgIsland reg genes).pdf]

(A)

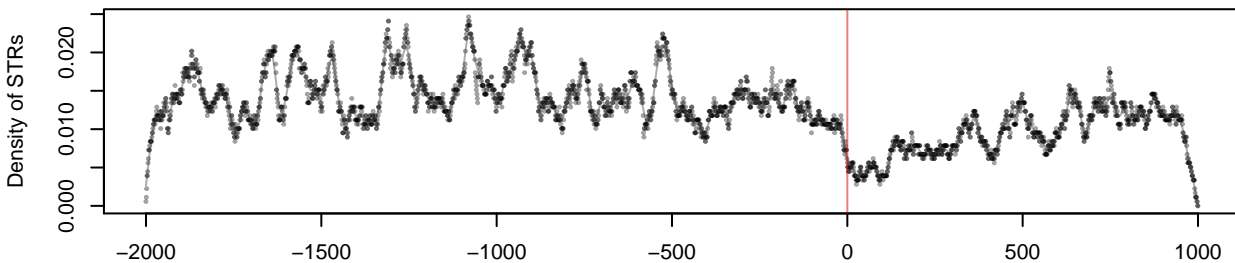

(B)

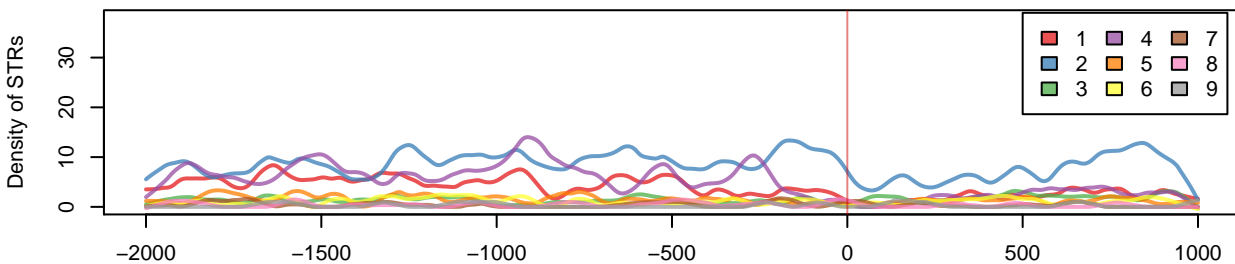

(C)

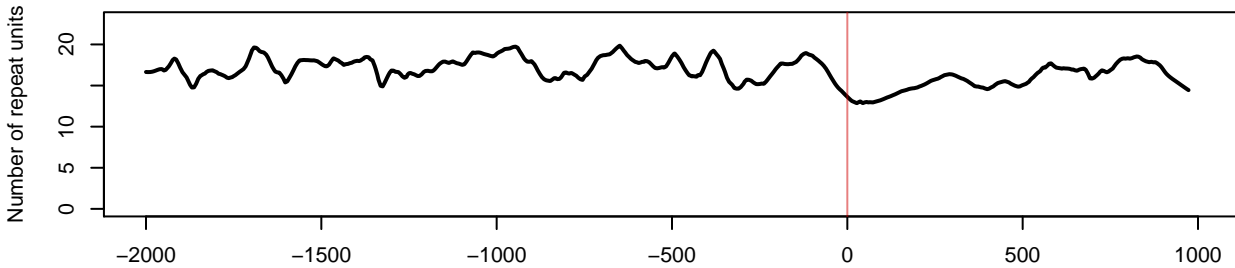

(D)

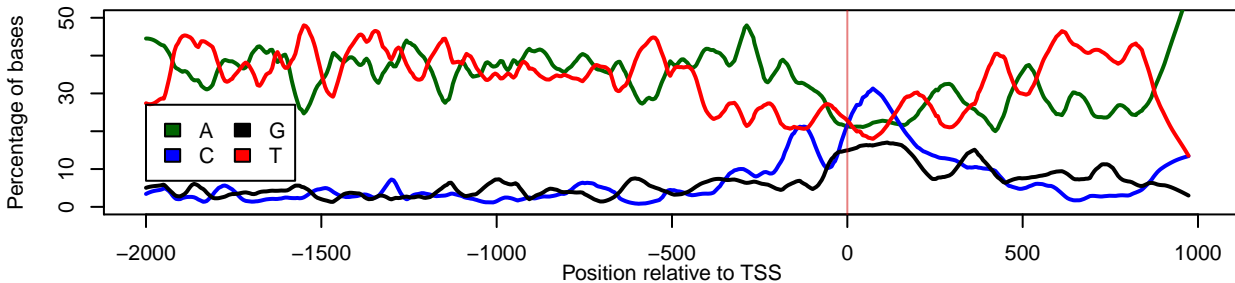

Supplement: Additional file 1 — Supplementary materials (Figures S1-S2; Tables S1-S3). [file 1471-2164-14-795-S1.zip › 1088845327100107_add1/Supp Fig2_(Non-CpgIsland reg genes).pdf]
