# Supplementary material for: STaRRRT: a table of short tandem repeats in regulatory regions of the human genome
Source: BMC Genomics. 2013 Nov 15;14:795. doi: 10.1186/1471-2164-14-795 (PMC3840602; doi:10.1186/1471-2164-14-795)
Supplement: Additional file 2 — Supplementary methods. (SQL code; R scripts; R/Markdown HTML-based report). [file 1471-2164-14-795-S2.zip › 1088845327100107_add2/STaRRRT_analysis_knitR-markdown.html]

STaRRRT Bioinformatics Analysis Document


# STaRRRT Bioinformatics Analysis Document

This is the R analysis document to accompany this paper:
**STaRRRT: A Table of Short Tandem Repeats In Regulatory Regions of the Human Genome**  
*Katherine A. Bolton, Jason P. Ross, Desma M. Grice, Nikola A. Bowden, Elizabeth G. Holliday, Kelly A. Avery-Kiejda and Rodney J. Scott*

## Generate distribution statistics

### All the files were loaded from this path: /home/jason/R\_SimpleRepeatsAroundTSS.

In the STaRRRT table there are 5264 elements in total. Of these, 0 rows originate from haplotype, unplaced chromosome chunks and random chromosome chunks. If required these are removed. For the complete 962714 row UCSC simpleRepeats table, 12449 rows originate from haplotype, unplaced chromosome chunks and random chromosome chunks and are removed. Similarly, in the complete 43284 row UCSC refGene table, 2277 rows originate from haplotype, unplaced chromosome chunks and random chromosome chunks and are removed.

```
if (sum(is_good_chrom_sr) != length(is_good_chrom_sr)) {
    sr <- sr[is_good_chrom_sr, ]
    rm(is_good_chrom_sr)
    is_str <- sr$period < 10
}
if (sum(is_good_chrom_tss) != length(is_good_chrom_tss)) {
    tss_sr <- tss_sr[is_good_chrom_tss, ]
    rm(is_good_chrom_tss)
}

if (sum(is_good_chrom_rg) != length(is_good_chrom_rg)) {
    refGene <- refGene[is_good_chrom_rg, ]
    rm(is_good_chrom_rg)
}
```

After filtering, there are 342258 short tandem repeats (STRs) in the simpleRepeats table.

The number of unique gene symbols in STaRRRT is 4441. The proportion of HGNC genes having a STaRRRT STR is 0.1878 and for unique refSeq transcripts this proportion is 0.1113.

**Table of percentage STaRRRT STRs per repeat period**

```
round(table(tss_sr$period)/nrow(tss_sr) * 100, 1)
```

```
## 
##    1    2    3    4    5    6    7    8    9 
## 23.9 28.6 10.1 14.2  7.4  6.1  3.5  2.9  3.3
```

**Table of the number of STaRRRT STRs per refGene transcript id**

```
starrrt_per_gene <- data.frame(table(tss_sr$refSeqId))
table(starrrt_per_gene$Freq)
```

```
## 
##    1    2    3    4    5    6 
## 3751  597   84   14    1    1
```

A list of the genes with three or more STaRRRT STRs

```
high_str_genes <- as.character(starrrt_per_gene$Var1[starrrt_per_gene$Freq >= 
    3])
sort(unique(tss_sr$hgncSymbol[tss_sr$refSeqId %in% high_str_genes]))
```

```
##   [1] "ANK2"            "ANXA2"           "AQP11"          
##   [4] "BCL9"            "BTBD2"           "C17orf98"       
##   [7] "C1orf85"         "C22orf15"        "C3orf55"        
##  [10] "C8orf37"         "CD1A"            "CHD3"           
##  [13] "CHRM2"           "CLDN3"           "COX6A1"         
##  [16] "CUX2"            "DCPS"            "DDX27"          
##  [19] "DHRS9"           "DHX57"           "DRP2"           
##  [22] "DUX2"            "ECM1"            "EIF2C4"         
##  [25] "EIF4A3"          "EMP2"            "EXT1"           
##  [28] "FAM90A1"         "FBXL21"          "FER1L4"         
##  [31] "FNDC8"           "FSCN3"           "GATA5"          
##  [34] "GATSL1"          "GATSL2"          "GUSBP11"        
##  [37] "IL28RA"          "IZUMO2"          "KCNK9"          
##  [40] "KCTD14"          "KDELR2"          "LECT1"          
##  [43] "LOC100093631"    "LOC100240735"    "LOC100506540"   
##  [46] "LOC284998"       "LUC7L3"          "LYZ"            
##  [49] "MACF1"           "MIR1292"         "MT1E"           
##  [52] "MYCBP2"          "MYT1L"           "NANOGNB"        
##  [55] "NAPSA"           "NOL6"            "NOP56"          
##  [58] "NPAS3"           "ONECUT2"         "PCSK5"          
##  [61] "PF4"             "PLEKHH2"         "PLSCR3"         
##  [64] "PPIAL4A"         "PRAMEF8"         "PRMT8"          
##  [67] "PRRT4"           "PTPRVP"          "PYGO1"          
##  [70] "RBMS1"           "RILPL2"          "RIMBP3"         
##  [73] "SCN1B"           "SEMA3A"          "SLC2A3"         
##  [76] "SLC52A1"         "SLFN12"          "SLITRK4"        
##  [79] "SMURF1"          "SPDYE2L"         "SPDYE8P"        
##  [82] "SPSB1"           "SRRM3"           "SSX2"           
##  [85] "STAT6"           "THAP11"          "TJP1"           
##  [88] "TNFSF12-TNFSF13" "TRIM5"           "TSG101"         
##  [91] "TTC24"           "TTLL13"          "TUSC5"          
##  [94] "UBE2Q1"          "WASF2"           "ZCCHC24"        
##  [97] "ZNF562"          "ZNF799"          "ZNF815P"        
## [100] "ZYG11B"
```

**Some refGene transcripts have 5-UTRs of length 0**

```
has_noutr <- refGene$utr_size == 0
table(has_noutr)
```

```
## has_noutr
## FALSE  TRUE 
## 36400  4607
```

**Some refGene transcripts have 5-UTRs less than 1 kb long**

```
table(refGene$utr_size < 1000)
```

```
## 
## FALSE  TRUE 
## 15029 25978
```

```
summary(refGene$utr_size)
```

```
##    Min. 1st Qu.  Median    Mean 3rd Qu.    Max. 
##       0      80     292    9890    4140 1880000
```

**The gene with the longest 5-UTR is**

```
refGene[refGene$utr_size == max(refGene$utr_size), c("name", "name2", "utr_size")]
```

```
##            name name2 utr_size
## 32157 NM_002839 PTPRD  1878880
```

## Figures

```
library(ggplot2)
library(GenomicRanges)

sr_period_props <- rbind(sr.table(sr = sr$period[is_str], name = "Period", id = "A) All STRs"), 
    sr.table(sr = sr$period[is_str & sr.gr %over% upstream.gr], name = "Period", 
        id = "B) Upstream"), sr.table(sr = sr$period[is_str & sr.gr %over% regregion.gr], 
        name = "Period", id = "C) Reg. region"), sr.table(sr = sr$period[is_str & 
        sr.gr %over% proximal.gr], name = "Period", id = "D) Prox. Promoter"), 
    sr.table(sr = sr$period[is_str & sr.gr %over% exon.gr], name = "Period", 
        id = "E) Exon"), sr.table(sr = sr$period[is_str & sr.gr %over% fiveutr.gr], 
        name = "Period", id = "F) 5'-UTR"), sr.table(sr = sr$period[is_str & 
        sr.gr %over% intron.gr], name = "Period", id = "G) Intron"), sr.table(sr = tss_sr$period, 
        name = "Period", id = "H) STaRRRT"))

hist_sr_period_props <- ggplot(sr_period_props, aes(x = factor(Period), y = Proportion, 
    fill = Dataset))
hist_sr_period_props + geom_bar(position = "dodge", stat = "identity") + theme_bw() + 
    scale_fill_grey() + xlab("Period")
```

**Panel showing the characteristics of STaRRT STRs over the regulatory region**

```
FigurePanel(tss_sr)
```

Most refGene transcripts have some overlap with a CpG island. These are captured and the previous figure is split into those regulatory regions with a CpG island overlap and those without.

```
table(regregion.gr %over% cpgIsland.gr)
```

```
## 
## FALSE  TRUE 
## 14052 26955
```

```
cpgisland_regregion_transcriptIds <- values(regregion.gr)$name[regregion.gr %over% 
    cpgIsland.gr]
is_cpg_reg <- tss_sr$refSeqId %in% cpgisland_regregion_transcriptIds
```

In the STaRRRT resource, most STRs are contained in regulatory regions overlapping CpG islands

```
table(is_cpg_reg)
```

```
## is_cpg_reg
## FALSE  TRUE 
##  1785  3479
```

**Panel of 3479 CpG island overlapping STaRRRT STRs**

```
FigurePanel(tss_sr[is_cpg_reg, ])
```

**Panel of 1785 non-CpG island overlapping STaRRRT STRs**

```
FigurePanel(tss_sr[!is_cpg_reg, ])
```

## HEAT Analysis

There are 5264 STRs in STaRRRT. In the UCSC simpleRepeat table there are 3287 STRs in exonic regions (of any purity). In intronic regions there are 134935 STRs. With a 90% purity filter this decreases to 94887.

For each STR in exons and introns a HEAT transcript ID is added.

```
str_exon.gr <- sr.gr[sr.gr %over% exon.gr & mcols(sr.gr)$period < 10]
exon_matches <- as.data.frame(exon.gr, stringsAsFactors = FALSE)[match(str_exon.gr, 
    exon.gr, match.if.overlap = TRUE), c("start", "end", "name", "name2")]
names(exon_matches) <- c("exonStart", "exonEnd", "refSeqId", "hgncSymbol")
str_exon <- cbind(as.data.frame(str_exon.gr, stringsAsFactors = FALSE), exon_matches)
names(str_exon)[1] <- "chrom"
str_exon$hinvId <- knownToHinv$hinvId[match(as.character(str_exon$refSeqId), 
    knownToRefgeneToHinv$refSeqId)]

str_intron.gr <- sr.gr[sr.gr %over% intron.gr & mcols(sr.gr)$period < 10 & mcols(sr.gr)$perMatch >= 
    90]
intron_matches <- as.data.frame(intron.gr, stringsAsFactors = FALSE)[match(str_intron.gr, 
    intron.gr, match.if.overlap = TRUE), c("start", "end", "name", "name2")]
names(intron_matches) <- c("intronStart", "intronEnd", "refSeqId", "hgncSymbol")
str_intron <- cbind(as.data.frame(str_intron.gr, stringsAsFactors = FALSE), 
    intron_matches)
names(str_intron)[1] <- "chrom"
str_intron$refSeqId <- as.character(str_intron$refSeqId)
str_intron$hgncSymbol <- as.character(str_intron$hgncSymbol)
str_intron$hinvId <- knownToHinv$hinvId[match(as.character(str_intron$refSeqId), 
    knownToRefgeneToHinv$refSeqId)]
```

The number of unique HGNC genes with exonic STRs is 2617 and the number of mapping HIT ids with exonic STRs is 2228. For the intronic STRs, the number of unique HGNC genes is 13361 and the number of mapping HIT ids with exonic STRs is 9300. There are 24972 unique HIT ids in total. The numbers of exonic STRs are comparable to STaRRRT HIT mappings, which makes comparison by p-value meaningful. However the large number of intronic STR HIT matches makes gene set enrichment comparison less meaningful. As introns are typically long and numerous, the observation of a single STR in a gene may not be particularly meaningful. Instead the strategy is to capture the genes with the highest density of intronic STRs.

```
# Number of introns per refSeqId
refseq_num_intron <- as.data.frame(table(as.character(mcols(intron.gr)$name)))
names(refseq_num_intron) <- c("refSeqId", "intron_num")
# Length of introns per refSeqId
refseq_length_intron <- data.frame(refSeqId = mcols(intron.gr)$name, intron_width = width(intron.gr), 
    stringsAsFactors = FALSE)
combined_intron_widths <- data.frame(tapply(refseq_length_intron$intron_width, 
    refseq_length_intron$refSeqId, sum))
names(combined_intron_widths) <- "intron_width"
combined_intron_widths$refSeqId <- rownames(combined_intron_widths)
str_intron_per_refseq <- data.frame(table(str_intron$refSeqId))
names(str_intron_per_refseq) <- c("refSeqId", "str_num")

str_intron_normalised <- merge(combined_intron_widths, str_intron_per_refseq)
str_intron_normalised$intron_str_density <- round(str_intron_normalised$intron_width/str_intron_normalised$str_num)
summary(str_intron_normalised$intron_str_density)
```

```
##    Min. 1st Qu.  Median    Mean 3rd Qu.    Max. 
##     262    7320   11200   14600   16700  582000
```

```
# Only use the refSeqIds that have introns with the highest quartile of STR
# density
high_intron_density_str_refseq <- str_intron_normalised$refSeqId[str_intron_normalised$intron_str_density < 
    quantile(str_intron_normalised$intron_str_density, 0.25)]
```

There are 3449 refSeq transcripts in the highest quartile of intronic STR density. This highest density quartile is composed of 17482 individual intronic STRs across 3444 HGNC genes.

Now HEAT tables are written and imported into the H-Invitational Database (H-InvDB) Enrichment Analysis Tool.

```
MakeHeatList(tss_sr, match_cutoff = 90, range_up = -2000, range_down = 1000, 
    knownToHinv = TRUE)
```

```
## 3258 unique hinvId elements in total.
```

```
MakeHeatControlList(str_exon, match_cutoff = 0, prefix = "exon", knownToHinv = TRUE)
```

```
## 2227 unique hinvId elements in total.
```

```
MakeHeatControlList(str_intron[str_intron$refSeqId %in% high_intron_density_str_refseq, 
    ], match_cutoff = 90, prefix = "intron", knownToHinv = TRUE)
```

```
## 2795 unique hinvId elements in total.
```

# Make 10 random intron samples

```
for (i in 1:10) {
    MakeHeatControlList(str_intron, match_cutoff = 90, sample = length(unique(tss_sr$hinvId)), 
        prefix = paste("intron_sample", i, sep = "_"), knownToHinv = TRUE)
}
```

```
## 9299 unique hinvId elements in total.
## Taking a sample of 3258.
## 9299 unique hinvId elements in total.
## Taking a sample of 3258.
## 9299 unique hinvId elements in total.
## Taking a sample of 3258.
## 9299 unique hinvId elements in total.
## Taking a sample of 3258.
## 9299 unique hinvId elements in total.
## Taking a sample of 3258.
## 9299 unique hinvId elements in total.
## Taking a sample of 3258.
## 9299 unique hinvId elements in total.
## Taking a sample of 3258.
## 9299 unique hinvId elements in total.
## Taking a sample of 3258.
## 9299 unique hinvId elements in total.
## Taking a sample of 3258.
## 9299 unique hinvId elements in total.
## Taking a sample of 3258.
```
